# Induction of Fish Biomarkers by Synthetic-Based Drilling Muds

**DOI:** 10.1371/journal.pone.0069489

**Published:** 2013-07-22

**Authors:** Marthe Monique Gagnon, Sajida Bakhtyar

**Affiliations:** Department of Environment and Agriculture, Curtin University, Perth, Australia; Indian Institute of Toxicology Reserach, India

## Abstract

The study investigated the effects of chronic exposure of pink snapper (*Pagrus auratus Forster*), to synthetic based drilling muds (SBMs). Fish were exposed to three mud systems comprised of three different types of synthetic based fluids (SBFs): an ester (E), an isomerized olefin (IO) and linear alpha olefin (LAO). Condition factor (CF), liver somatic index (LSI), hepatic detoxification (EROD activity), biliary metabolites, DNA damage and stress proteins (HSP-70) were determined. Exposure to E caused biologically significant effects by increasing CF and LSI, and triggered biliary metabolite accumulation. While ester-based SBFs have a rapid biodegradation rate in the environment, they caused the most pronounced effects on fish health. IO induced EROD activity and biliary metabolites and LAO induced EROD activity and stress protein levels. The results demonstrate that while acute toxicity of SBMs is generally low, chronic exposure to weathering cutting piles has the potential to affect fish health. The study illustrates the advantages of the Western Australian government case-by-case approach to drilling fluid management, and highlights the importance of considering the receiving environment in the selection of SBMs.

## Introduction

In Western Australia, the environmental acceptability of a drilling activity is assessed by government on a case-by-case basis with the likelihood and consequence of the environmental impact determining the acceptability of the activity. In this regard, the use of particular drilling muds is not approved per se, but is considered in the context of the whole drilling application [1]. The environmental performance assessment criteria considered in the use of particular drilling muds includes acute and chronic toxicity testing, biodegradation rate and bioaccumulation potential.

Mineral oil-based drilling muds have excellent technical attributes but poor environmental performance because of their tendency to persist in cutting piles. The introduction of synthetic based drilling muds (SBMs) in the early 1990s reduced the risk to the environment whilst providing the technical qualities required for drilling under difficult situations. However, recent observations of SBMs cutting piles on the seabed offshore Western Australia have indicated that the cutting piles are more persistent than expected [2]. In addition, investigations into degradation of drilling muds have shown that synthetic based drilling muds can resist degradation for long periods of time [3]–[4]. There is a need to further consider if chronic exposure to weathering drilling muds has the potential to adversely affect fish health.

It is now accepted that chronic toxicity testing is more relevant to environmental management than acute toxicity results [5]–[6] as the aquatic biota will be exposed in the longer term to the cutting piles containing up to 10% drilling muds. In addition, results from acute toxicity do not always relate to safe chronic exposure levels. For example when exposure to drilling muds increases from 2–4 days to 10–30 days, the concentration of drilling muds that cause lethal effects in fish fry decrease by 2–3 orders of magnitude [7]. While acute toxicity testing suggests that SBMs are practically non-toxic, the chronic toxicity of weathering cutting piles remains largely unknown.

The present study was undertaken to investigate the potential health effects of chronic exposure to three types of SBFs on fish health. Juvenile fish were exposed to an ester-based (E), or to an isomerised olefin-based (IO), or to a linear alpha olefin-based (LAO) drilling fluid for 28 days under laboratory conditions. Biomarkers of exposure (ethoxyresorufin-*O*-deethylase [EROD] activity and biliary metabolites) and biomarkers of effects (condition factor, liver somatic index, DNA damage and stress proteins) were measured at the end of the exposure period. These biomarkers have shown to be amongst the most relevant to identify impacts of xenobiotics on organisms [8]–[10]. The suite of biomarkers provides an indication of the potential impacts of exposure to drilling fluids on fish health, and assist environmental managers in the selection of environmentally acceptable drilling fluids.

## Materials and Methods

### Ethics Statement

This study was carried out in strict accordance with the recommendations in the Western Australian Animal Welfare Act 2002 [11] and the Australian code of practice for the care and use of animals for scientific purposes 7th edition 2004 [12]. The protocol was approved by the Curtin University Animal Ethics Committee (Approval Number N54-07). All efforts were made to minimize suffering.

### Fish Exposure

Juvenile pink snapper (*Pagrus auratus Forster 1801*, 8 months old) from a local hatchery were intentionally selected as reproductive hormones have the potential to interfere with some biomarker determination. Fish (N = 8 per treatment) were acclimatised for 14 days in 100 L aquariums prior to the introduction of the drilling fluids. The average loading in each aquarium was approximately 4.5 g of fish body weight litre^−1^ seawater. The seawater used for the daily 50% water renewal was collected in the proximity of the local hatchery of origin so the laboratory seawater had similar physicochemical characteristics as the water the fish were used to. The water parameters salinity, pH and temperature were measured daily however the oxygen could not be measured due to the weathering drilling fluids clogging the oxygen probe upon contact and damaging it irreversibly. The aquariums were continuously aerated. Fish were fed at a maintenance ratio of 1% body weight/day during the acclimation and experimental periods. The basic procedures followed according to the method of standard handling and water renewals [13].

Two control groups were an integral part of the experiment. A negative control group was not exposed to drilling fluids, while a positive control group was injected with benzo(a)pyrene (B(a)P) at a dose of 1.0 µg chemical/g fish. The objective was to trigger specific biochemical responses (EROD activity and bile metabolites) in order to ensure the biochemical responsiveness of the fish. The positive control group was killed 4-days post-injection as the biomarker response has been shown to be maximal at 4 days following injection with this compound [14].

### Synthetic Based Drilling Muds

Three synthetic based entire drilling mud systems were used. These were ester-based generic chemical structure CH3– (CH2) n – CO – O – (CH2) n – CH3, IO – based generic chemical structure CH3– (CH2) n – CH = CH – (CH2) n – CH3 and LAO-based generic chemical structure CH3– (CH3) n – CH = CH2 were used for the present study. Three SBMs were mixed in the laboratory specifically for the experiment and were not drawn from existing batches. It is therefore assumed that drilling muds were not contaminated by petroleum hydrocarbons. The ingredients of the drilling muds were identical expect for the base fluids (E base oil, IO base oil and LAO base oil) differed amongst muds.

The muds were gently deposited on the bottom of the aquarium via a 30 mm-diameter hose. The drilling muds settled on the bottom of the aquariums and did not readily mix in the water column. The same mud was left undisturbed during the daily water changes, for the duration of the exposure period (28 days).

### Chemical Analysis

Water samples from Ester-based, IO-based and LAO-based SBMs exposure tanks were collected in duplicate on weekly basis for determination of pollution levels in the water along with the negative control treatment (unexposed to SBMs). A suite of chemical analyses such as Total Recovered Hydrocarbons (TRH), Chemical Oxygen Demand (COD) and trace heavy metal analysis were done on all water samples.

TRH were extracted according to the methodology adapted from the US EPA method 3510 and the APHA-AWWA-WEF method 5520 F [15]–[16]. Briefly, hydrocarbons from water samples were extracted with dichloromethane (DCM) by using sodium sulphate columns. A hexane exchange and silica gel clean up was done on all samples as silica gel has the ability to adsorb polar materials and leaves the hydrocarbons in the extract. Extracts were concentrated to a known volume and injected into a Gas Chromatograph - Flame Ionization Detector (GC-FID) along with standards. The individual components of hydrocarbon’s range from C_10_–C_36_ were separated with a non-polar capillary column. Detection was achieved by flame ionization. TRH concentrations were determined through electronic integration by comparison with calibration standards. The acceptable spike recovery for TRH C_10_–C_36_ region is 50 - 150%. While acceptable Relative Percentage Difference (RPD) is 60%. Limit of Reporting (LOR) is <250 µg/L. Results are recorded in µg/L.

Chemical Oxygen Demand (COD) determination is often used to measure the pollutants in waste water. COD values of Ester-based, IO-based and LAO-based and SBMs-exposed water samples were determined according to closed reflux colorimetric APHA-AWWA-WEF method 5220 D [17]. The water samples had high salinity (Table 1) therefore; all samples were diluted ten times with deionized water in order to avoid any chloride interference with the COD method. Excess quantities of potassium dichromate and sulphuric acid reagent were used to reflux the samples. COD was measured on colorimeter at 600 nm.

**Table 1:** Physicochemical parameters of the seawater during the acclimation (n = 14 days) and experimental (n = 28 days) periods.

Treatment	Salinity (ppt)	pH	Temperature (°C)
Negative control	35.6±0.25	8.29±0.01	16.7±0.20
E	35.8±0.18	8.29±0.02	16.6±0.21
IO	35.8±0.12	8.26±0.01	16.7±0.15
LAO	35.8±0.19	8.30±0.02	16.6±0.14
Positive control	35.4±0.21	8.29±0.02	16.6±0.20

Heavy metals in the three SBFs exposed water samples were determined by Inductively Coupled Plasma Atomic Emission Spectroscopy (ICP- AES) according to US EPA Method 200.7 [18]. Samples were digested with dilute mineral acids HCl and H_2_NO_3_ and diluted five times with deionized water to avoid potential interferences and to determine concentrations beyond the linear calibration range due to high salinity of samples. The samples were run through ICP and concentrations of multi elements Al, Ba, Be, B, Cd, Cr, Co, Cu, Fe, Pb, Mn, Mo, Ni, Sn, Ag, V and Zn were recorded in mg/L. Determination of As and Se in water samples were done through Inductively Coupled Plasma optical Emission Spectrometer using hydride generation technique (ICP- OES). Water samples for Hg were analysed by a method adopted from revised US EPA method 245.2 [19]. Water samples were digested with mineral acids and mercury concentrations were determined by Cold Vapour Atomic Absorption Spectrophotometry. The limits of reporting (LOR) for all elements are reported with the results.

### Biomarker Determinations

The fish were weighed and standard fork and total lengths were recorded. An external examination was conducted for abnormalities. A sample of blood was taken from the caudal vein using a vaccutainer. The blood samples were allowed to clot on ice for 15 minutes and immediately centrifuged for 10 minutes at 3000 rpm at 4°C. Each fish was killed by the method of Ike Jime (spike through the brain), dissected and the bile collected from the gall bladder using a 1-mL syringe and needle. Livers were removed, quickly examined for any anomaly, weighed, rinsed in ice-cold KCl, and a 1-g sample taken for analysis. All samples were immediately placed in liquid nitrogen then transferred to a freezer and held at –80°C until analysis. Gonads were removed and examined for anomalies then weighed. The gonads and remaining abdominal organs were discarded and the fish weighed to record the carcass weight.

The condition factor of the fish was calculated as the [carcass weight/(fork length)^3^]×100, and gives an indication of the ‘fattiness’ of the animal. The liver somatic index was calculated as (liver weight/carcass weight)*100, and gives an indication of the size of the liver relative to the body size. The carcass weight was used rather than the total weight as carcass weight (total weight minus viscera) as it eliminates variation due to food presence in the stomach and intestines.

### EROD Assay

EROD activity was measured using a standard protocol [20]. Each liver sample was thawed on ice then homogenised in HEPES pH 7.5 using a Heidolph DIAX 900 homogeniser. The homogenate was centrifuged (Jouan CR3i centrifuge) at 9000 g for 20 mins at 4°C and the S9 post-mitochondrial supernatant (PMS) collected for immediate use. The reaction mixture contained HEPES pH 7.8, MgSO_4_, BSA, NADPH solution and PMS. The reaction was initiated by adding ethoxyresorufin, incubated at room temperature for 2 mins, and the reaction terminated by adding methanol. Resorufin standards (0.000 to 0.085 M) and samples were centrifuged to precipitate proteins and the fluorescence of the supernatant was immediately read on a Perkin-Elmer LS-5 Luminescence Spectrometer at excitation/emission wavelengths of 535/585 nm (slit 10 ex/10 em). Protein content of the PMS was determined according to [21]. EROD activity was expressed as picomoles of resorufin produced, per mg of total protein, per minute (pmol R/mg Pr/min).

### Bile Metabolites

It is possible that some water-soluble ingredients of the SBF are assimilated by the fish. Metabolisation of these compounds can lead to the presence of metabolites in the bile which have the potential to interfere with the reading of PAH metabolites in field-collected animals, with the PAHs originating from the petroleum compounds contained in the discharged cuttings. The presence of biliary chemicals fluorescing at the PAH wavelengths in SBF-exposed animals does not imply that these chemicals are PAH metabolites of petroleum origin. Rather, it only points to the presence of unidentified chemicals fluorescing at the PAH wavelengths. Therefore, the presence of metabolized compounds in the biliary secretions has been included in the present study to assess if compounds originating from the drilling fluids appeared in the bile, which could potentially interfere with the PAH determinations routinely done in field sampling. If the biliary secretions from fish exposed to the various drilling fluids show no interference with PAH metabolites, then this biomarker will be able to discriminate exposure to drilling fluids from exposure to drill cuttings containing petroleum compounds.

The biliary metabolite determination was performed by fixed fluorescence (FF) measurement [22]. The method is semi-quantitative, and reports metabolised chemicals fluorescing at the naphthalene, pyrene or benzo(a)pyrene [B(a)P] specific excitation/emission wavelengths. Fluorescent readings were performed at the naphthalene excitation/emission 290/335 nm using 1-naphthol (Sigma) as a reference standard. Metabolites fluorescing at the pyrene and B(a)P wavelengths were measured using 1-hydroxypyrene as a reference standard at 340/380 nm and 380/430 nm for pyrene and B(a)P wavelengths, respectively. Metabolites fluorescing at the naphthalene wavelength are reported in mg of 1-naphthol fluorescence units equivalent per mg biliary protein, and those fluorescing at the pyrene and B(a)P wavelengths are reported in µg of 1-OH pyrene fluorescence units equivalent per mg biliary protein. Therefore, the biliary metabolite levels measured represent fluorescence-equivalents of PAH metabolites used as standard.

Bile samples were thawed on ice and diluted to 1∶2000 in 50% HPLC grade methanol/H_2_O for determination of metabolites fluorescing at the pyrenol and B(a)P wavelengths. The bile was further diluted to 1∶5000 for the determination of metabolites fluorescing at the naphthalene wavelength. The fluorescence reading of bile was converted to 1-naphthol or 1-OH pyrene equivalents from the linear regression curves. A previous study [23] has shown that the normalisation for protein concentration in the bile can, to a large extent, account for changes in the level of biliary metabolites due to differences in the feeding status of some fish. The protein content of the bile reflects the amount of water in the bile, or the dilution of the bile, when collected from the gall bladder. Bile was diluted in 19 volumes of double distilled H_2_O (bile : water 1∶20) and the protein content determined using standard methods [21]. Biliary metabolites are reported on the basis of biliary protein (metabolite/mg protein).

### DNA Damage

The measurement of DNA damage was performed by the alkaline unwinding assay using liver tissue [24]. Briefly, the tissue was hand homogenized with DNAzol® and centrifuged at 8000 g for 10 minutes at 5°C. Ethanol was added to the isolated supernatant to precipitate the DNA. The isolated DNA was cleaned using a Tris-EDTA buffer, and the double-stranded (DS), single-stranded (SS) and partially unwounded (DSS) DNA were obtained by treating samples with NaOH and incubating at various temperatures [24]. The fluorescence of each sample was measured using a Perkin-Elmer LS-5 Luminescence Spectrometer at excitation/emission wavelengths of 350/453 nm (slit 5 ex/10 em), and the F-value (representing DNA integrity) was calculated as F = (DSS–SS)/DS–SS.

### Stress Proteins

Stress protein (HSP-70) response was measured using standard electrophrosis protocols optimized for *Acanthopagrus butcheri* [25]. Gill tissue was weighed and homogenized with Tris-PMFS buffer using a Heidolph DIAX 900 homogeniser. The homogenate was centrifuged at 12000 g for 98 min at 4°C. Proteins were determined in the supernatant [21]. Supernatant containing 40 µg proteins was mixed with sample buffer (Bio-Rad Laboratories, NSW, Australia) at a ratio of 1∶2 supernatant/buffer then placed in a waterbath at 95°C for 4 min. Samples were loaded in duplicate into 12% Tris-Glycine iGels (Life Therapeutics, NSW, Australia) wells with heat shock standardized controls loaded into the two outermost wells. The gels were run at 225V, 120 mA (60 mA per gel) for 40 min in a mini-Protean 3 electrophoresis cell (Bio-Rad).

Proteins were transferred from iGels to 0.2 µm supported nitrocellulose membranes in a mini Trans-Blot electrode module (Bio-Rad) at 100 V, 250 mA for 1 hour. Following Western transfer the blots were blocked in 5% skim milk powder dissolved in Tween-phosphate buffered saline on a shaker for 1 hour. The blots were probed overnight at 4°C with monoclonal (mouse) anti-heat shock protein 70 antibody (IgG1, Bio-Scientific, Gymea, Australia), diluted 1∶5000 in Tris buffered saline (TBS), then the secondary antibody (goat anti-mouse IgG peroxidase conjugated, Progen Bioscience, Archerville, Australia) was applied, diluted 1∶30000 in Tween-TBS (TTBS) and allowed to incubate for 2 hours. Between each step, the blots were washed three times with TTBS then finally washed in TBS to remove the Tween.

A working solution of chemiluminescent substrate was prepared using the Super Signal® West Pico Chemiluminescent Substrate kit (Progen Bioscience). Under dark conditions, each blot was wetted with the chemiluminescent solution then exposed to a radiographic film (CL-Xposure™ X-Ray film, Progen Bioscience) which was immediately developed. The films were scanned and the pixel density of the images analysed using NIH image program. The bands on different blots were calibrated to known standards to enable comparison between blots. HSP-70 levels are reported as pixels per µg total protein (µg HSP-70 µg pr^−1^).

### Statistical Analysis

Physiological parameters total and carcass weight, fork length, condition factor (CF) and liver somatic index (LSI) as well as biomarker levels were compared between treatments using a one-way analysis of variance (ANOVA) after verification of normal data distribution and homoscedasticity. Post-hoc comparisons were performed with the Dunnett’s test, which compares one negative control group to all other groups. For all statistical testing, a significance level alpha (α) of 0.05 was applied.

## Results

The physicochemical water parameters were stable throughout the experiment (Table 1). Initially, the fluids deposited at the bottom of the aquarium settled rapidly and were apparently not water soluble. With weathering, a residue appeared at the surface due to the separation of the synthetic oil from other ingredients, and the bottom residue appeared to flocculate. The ester-based fluid separated more than the other drilling fluids and caused a thick (1 mm) residue at the surface of the aquarium. The surface scum and the bottom sediments were not disturbed during water changes.

### Chemical Analysis

TRH analysis revealed that water samples collected from tanks exposed with LAO-based SBF have demonstrated comparatively higher values of hydrocarbons (7,700,000 µg/L) than IO-based and E-based SBMs (3,350,000 µg/L and 74,000 µg/L respectively). IO-based SBF showed highest TRH value in week three whereas LAO-based and E-based SBM exposure tanks showed highest TRH reading in week two. Water samples collected from the negative control aquariums showed minimal increase in TRH results from week zero to week four ranging from <250 µg/L to 1300 µg/L (Table 2). The limits of reporting (LOR) for all elements are reported in mg/L in Table 2.

**Table 2:** Analysis of water samples of Ester, Internal Olefins and Linear Alpha Olefins based SBMs and Negative Control treatments in which juvenile pink snappers were exposed.

		TRH	COD	Al	Ba	B	Fe	Mn	Mo	Zn
	Units →	µg/L	mg/L	mg/L	mg/L	mg/L	mg/L	mg/L	mg/L	mg/L
	LOR →	<250	<50	<0.005	<0.001	<0.010	<0.005	<0.001	<0.005	<0.005
**Negative Control**	Wk 0	<250	<50	0.02	0.016	5.15	0.020	0.003	<0.005	0.016
	Wk 1	<250	<50	0.011	0.011	6.05	0.013	0.002	<0.005	0.027
	Wk2	510	<50	0.017	0.012	5.85	0.021	<0.001	<0.005	0.036
	Wk 3	945	<50	<0.005	0.01	6.2	<0.005	<0.001	<0.005	0.039
	Wk 4	1300	<50	<0.005	0.011	5.55	<0.005	<0.001	<0.005	0.046
**Ester**	Wk 0	<250	90	0.031	0.27	5.85	0.026	0.023	<0.005	0.014
	Wk 1	300	180	<0.005	0.37	5.35	<0.005	<0.001	0.011	0.008
	Wk2	74500	2550	0.023	0.676	6.3	0.024	0.011	0.011	0.007
	Wk 3	19900	1000	<0.005	0.356	5.75	<0.005	0.007	<0.005	0.018
	Wk 4	38000	580	0.145	0.015	5.1	<0.005	<.001	0.014	0.013
**Internal Olefins**	Wk 0	3750	n.a	0.025	0.16	5.05	0.018	0.006	0.013	0.027
	Wk 1	31500	230	0.028	0.555	5.05	0.019	0.018	0.009	0.056
	Wk2	1215000	2415	0.031	0.134	6.05	0.046	0.039	<0.005	0.018
	Wk 3	3350000	2150	0.025	0.123	5.5	0.025	0.018	0.01	0.025
	Wk4	245000	360	0.02	0.075	5.55	0.025	0.015	0.015	0.02
**Linear Alpha Olefins**	Wk 0	3900	n.a	0.031	0.22	5.35	0.027	0.023	0.090	0.014
	Wk 1	155000	260	0.012	0.34	5.45	0.015	0.014	0.090	0.019
	Wk2	7700000	6600	0.0235	0.061	5.75	0.014	0.029	0.011	0.025
	Wk 3	1845000	1300	0.025	0.011	5.75	0.016	0.01	0.015	0.035
	Wk 4	1665000	2550	0.024	0.049	5.45	0.015	0.015	0.009	0.036

COD results demonstrated that all the three exposed SBMs have shown maximum COD values in week two analysis, with LAO-based SBM having 2.5 times higher COD (6600 mg/L) relative to IO-based and E-based CODs (2415 mg/L and 2550 mg/L respectively). COD in unexposed negative control group remained below the limit of reporting for the duration of exposure period; that is <50 mg/L (Table 2).

Heavy metal analysis demonstrated that water samples collected from the tanks exposed with three SBFs as well as unexposed negative control group have shown results below the specified limits of reporting (Table 2) for trace elements Ag, As and Be, Cd, Cr, Co, Cu, Ni, Se, V, Pb, Sn and Hg. Al was slightly lower (<0.005–0.020 mg/L) in the negative control tank relative to all other treatments, which were all in the range of <0.005–0.016 mg/L. Ba was also lower (0.010–0.016 mg/L) in the negative control tank tanks. However, its concentration increased from IO (0.075–0.160 mg/L) to LAO (0.049–0.340 mg/L) to E (0.015–0.676 mg/L). All SBMs exposed water samples along with negative control group showed high Boron readings (5.1–6.3 mg/L). Iron was detected in SBM and Negative control samples in range <0.005–0.027 mg/L except IO week 2 samples which showed slightly high iron values (0.046 mg/L). Low concentrations appeared for Mn, Mo and Zn that were not significantly different to negative control samples except Mo in all negative control samples was below the limit of reporting (Table 2).

### Physiological Parameters

Fish were of similar size, as measured by total weight and fork length, in all treatments (p>0.05) (Table 3). The CF was significantly higher (p = 0.009) in fish exposed to the ester-based fluid relative to all other groups. Exposure of juvenile pink snapper to the ester-based fluid caused liquid retention in the abdominal cavity. LSI was significantly (p = 0.007) higher in the ester-based mud exposed fish, relative to the negative control group (Table 3).

**Table 3:** Physiological parameters for juvenile pink snapper exposed to ester, internal olefins, and linear alpha olefins-based drilling muds for 28 days.

Treatment	Total Weight (g)	Fork Length (cm)	CF	LSI
Negative Control	5.90±0.18	5.95±0.08	1.65±0.03	0.95±0.03
Ester	5.74±0.26	5.59±0.11	1.89±0.06*	1.26±0.06*
Internal Olefins	5.52±0.32	5.57±0.10	1.77±0.04	1.16±0.06
Linear Alpha Olefins	4.91±0.28	5.55±0.12	1.66±0.05	1.14±0.07
Positive Control	5.25±0.26	5.58±0.10	1.65±0.03	1.01±0.04

N = 8 per treatment.

* indicates a significant difference (p<0.05) relative to negative control group.

### Biomarkers

**Figure 1:**
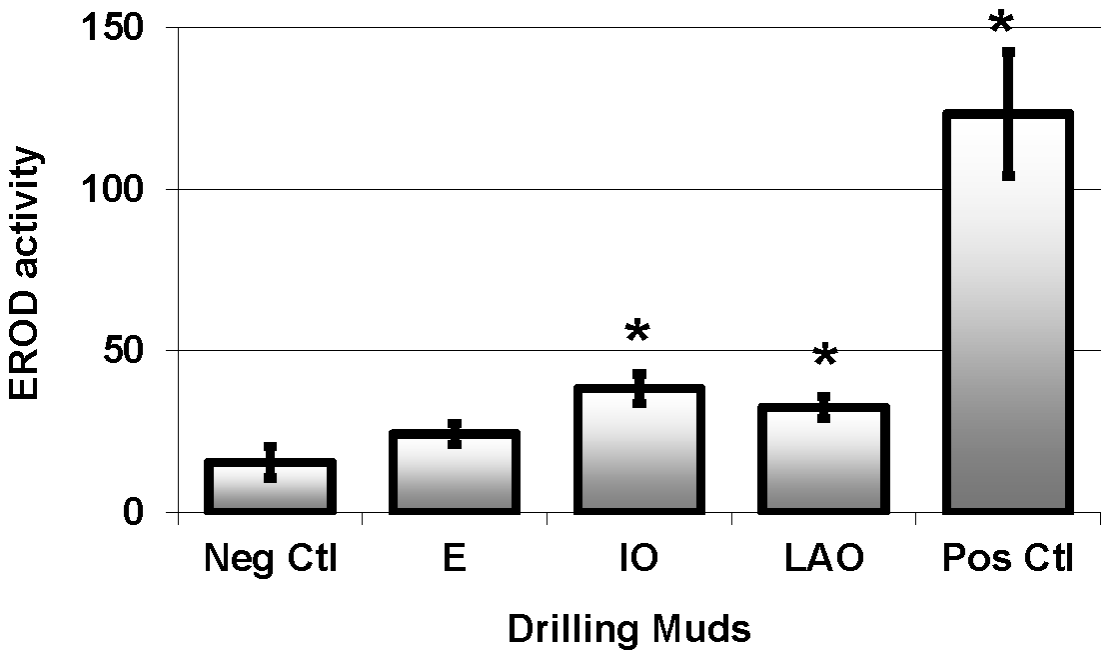
EROD activity (pmol resorufin/mg pro/min, mean ± SEM) in pink snapper exposed to 5% v/v synthetic-based drilling muds for 28 days. * indicates statistically different from negative control fish.

The negative control group demonstrated the lowest EROD activity level, while the positive control group injected with a potent EROD inducer had the highest activity level (p<0.00). While fish exposed to the ester-based fluid had similar EROD activity to the negative control group, fish exposed to the IO and LAO treatments had significantly higher (p<0.00) EROD activity relative to the unexposed fish (Figure 1).

**Figure 2:**
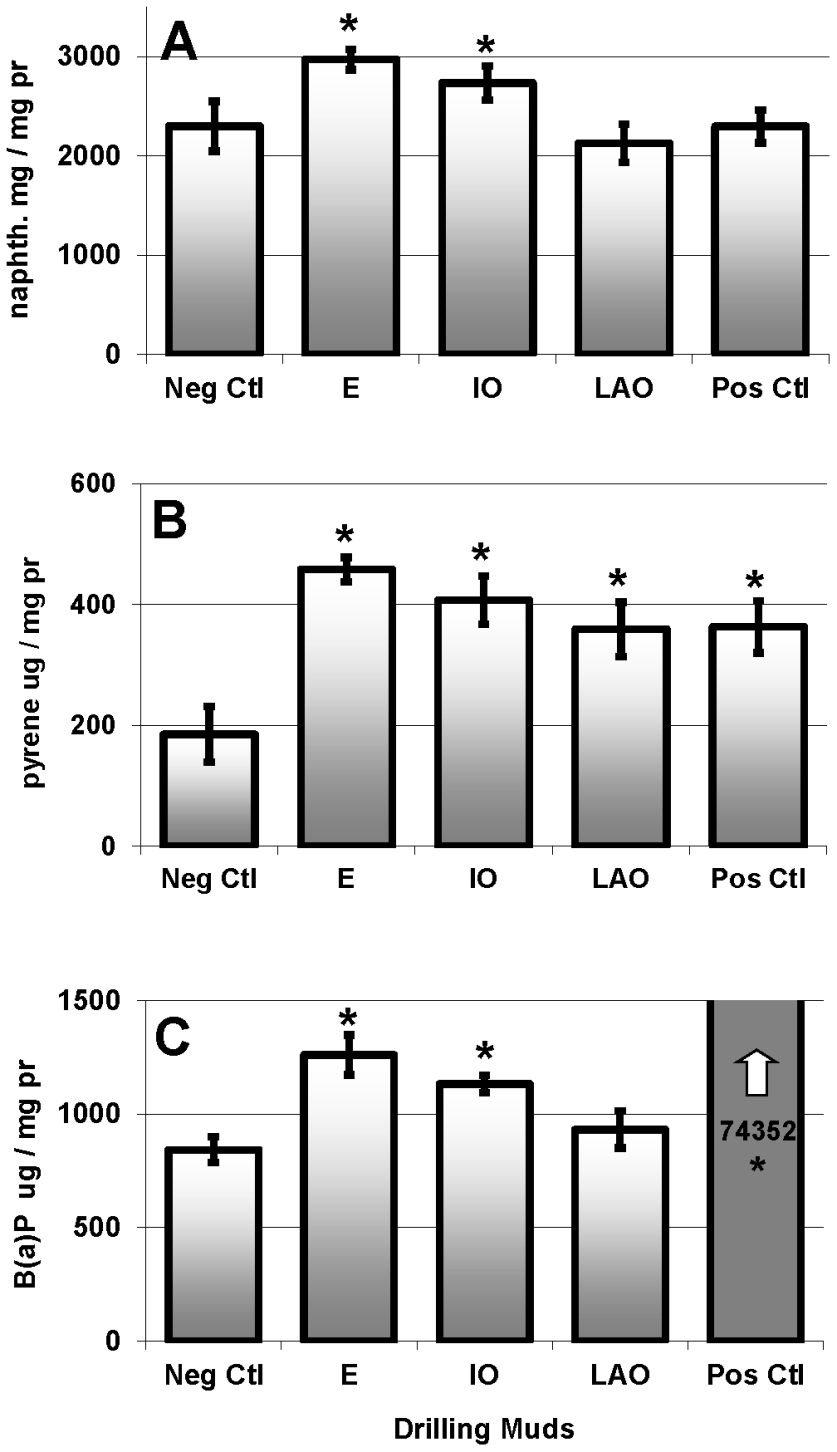
Biliary fluorescence (mean ± SEM ) at A: naphthalene wavelength, B: pyrene wavelength, C: B(a)P wavelength. E: ester; IO: isomerised olefin; LAO: linear alpha olefin SBMs. * indicates statistically different from negative control fish.

Fish exposed to the E-based, and to the internal IO-based fluids accumulated biliary metabolites fluorescing at the naphthalene and pyrene wavelengths, but not at the B(a)P wavelength. Fish exposed to the LAO-based fluid accumulated biliary metabolites measured at the pyrene wavelengths only (p<0.05). The positive control fish injected with B(a)P had significantly (p<0.00) higher levels of biliary metabolites fluorescing at the B(a)P wavelength relative to the negative control group, demonstrating that the liver detoxification process was effective and that metabolites did accumulate in the bile (Figure 2 A–C).

**Figure 3:**
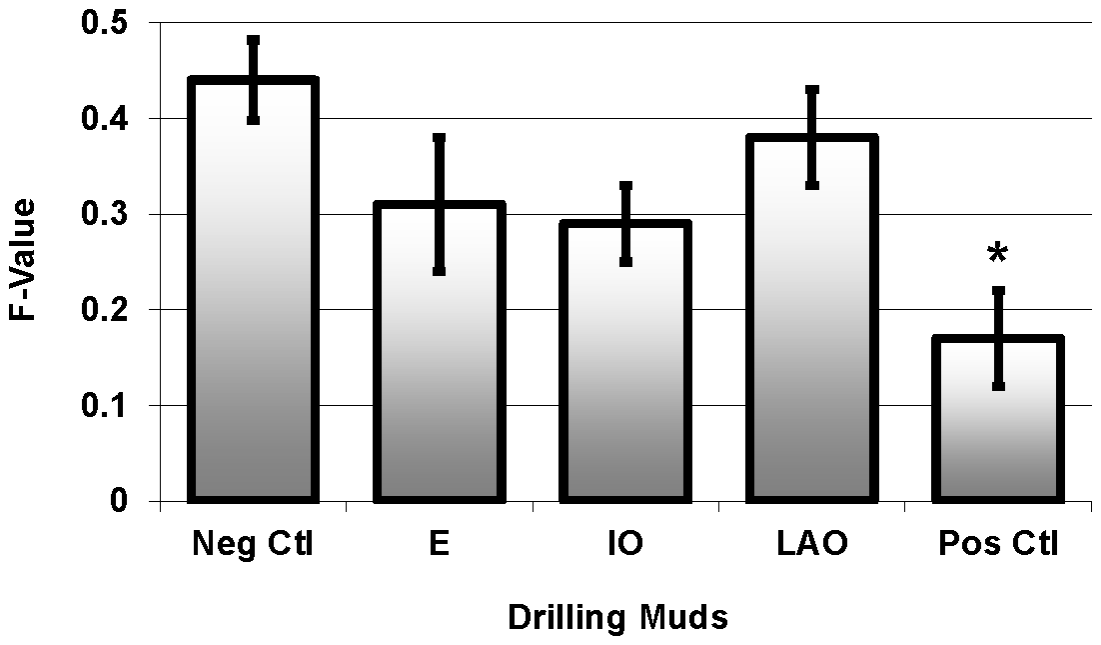
DNA damage (F-value, mean ± SEM) measured in pink snapper exposed to E: ester; IO: isomerised olefin; and LAO: linear alpha olefin SBMs. * indicates statistically different from negative control fish.

Exposure to the synthetic based drilling fluids did not result in increased DNA damage (p = 0.123) when treated fish were compared to the negative control group Figure 3).

**Figure 4:**
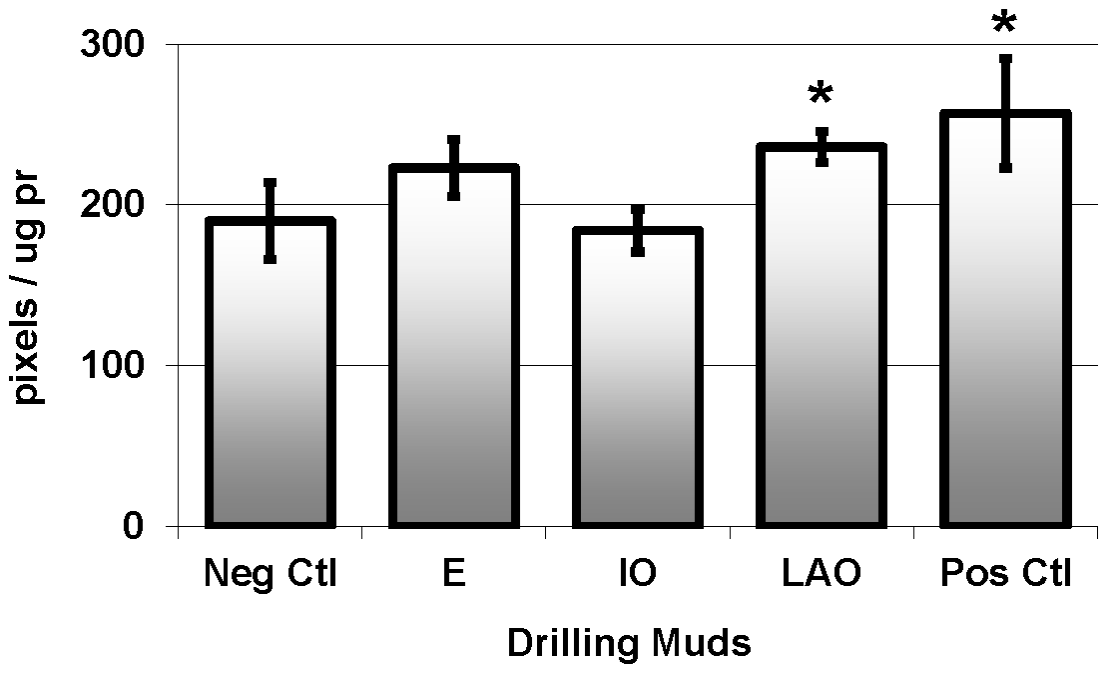
Stress proteins HSP-70 (pixels/mg pr, mean ± SEM) measured in pink snapper exposed to E: ester; IO: isomerised olefin; and LAO: linear alpha olefin SBMs. * indicates statistically different from negative control fish.

Only the fish exposed to LAO expressed significantly (p = 0.033) higher levels of HSP-70 in the gills, relative to the negative control group (Figure 4).

## Discussion

The major wastes generated during the drilling of petroleum exploration and production wells are drill cuttings mainly composed of crushed sedimentary rocks. In Western Australia, and subject to approval by regulators, drill cuttings can be discharged on the ocean floor with up to 10% (by weight) adhering drilling fluids [2]. The volume of drill cuttings discharged on the seafloor is approximately 1000 m^3^ per well [26] but can be as high as 20000 m^3^ [1]. Cutting piles might therefore contain significant amounts of drilling fluids which will weather slowly under the cold, often anaerobic conditions.

Synthetic based ester muds are lower molecular weight compounds and biodegrade easily relative to IO and LAO [27]. LAO are comprised of carbon chain length C_8_– C_20._ Most common LAO drilling fluids are composed of C_14_C_16_ with a double bond on terminal or alpha position. The commonly used IO are branched molecule with C_16_C_18_ chain. LAO and IO may have similar molecular weight but small difference as in molecular structures can give them different properties. Degradation is initiated by oxidation of double bond and branching can interfere with the oxidation [28]. High branch and high molecular weight slow down the biodegradation of the molecule.

Hydrocarbon and COD results from chemical analysis revealed that ester-based SBF samples showed less TRH values being a smaller molecule relative to two other groups and maximum degradation occurred in week second. TRH and COD readings for IO demonstrated that being a branched molecule degradation rate was slow and degradation continued for the exposure period. High values of COD also reflect the chemical oxygen demand by oxidation of compounds. SBM accumulation and high degradation may cause oxygen depletion in the sediment near the discharge sites [29].

Recent studies revealed that eminent concentration of barium and hydrocarbons were detected in sediments within 500 m of cutting discharge and benthic fauna were altered most likely from organic enrichment of SBM [29]. Detection of heavy metals in the present study demonstrated that although the concentrations of recalcitrant is not significantly high but repeated discharge of SBMs may lead to accumulation of these elements in the marine sediment which may reduce the ability of marine environment to support a diverse community of organisms.

The synthetic oil based drilling muds have been classified by laboratory-based acute lethality tests as practically non-toxic, with 5-day LC50 greater than 20000 mg/kg for the marine Australian native mollusc *Paphies elongate* [30]. The majority of field studies that observed impacts from cutting discharges attributed the changes in bottom invertebrate communities to increased biological oxygen demand and alteration of the sediment texture [27], [31], or to the presence of hydrocarbons in the cutting piles [32]. Contrary to acute toxicity testing results, our study has shown that chronic exposure of fish to SBMs has the potential to affect fish health, as evidenced by induced biomarkers.

EROD activity induced by exposure to IO and LAO, and accumulation of biliary metabolites represent biomarkers of exposure to bioavailable contaminants. It was not expected that EROD activity would be induced as the molecular shape of synthetic base mud does not conform to the classic EROD inducers such as B(a)P. However other ingredients of the drilling muds, or their metabolites, might be responsible for the weak induction observed. No known adverse health effects are directly associated to EROD induction or biliary metabolite accumulation, these biomarkers are therefore considered biomarkers of exposure. However, increases in CF and LSI as well as the high stress protein level following exposure of fish to the ester-based fluid has the potential to have significant effects on the health of fish. Especially significant is the retention of clear liquids in the abdominal cavity of the juvenile pink snapper exposed chronically to ester-based muds. This condition resulting in an increased CF has not been observed in fish exposed to other drilling muds. In the breakdown of ester-based drilling fluids, the likelihood of breaking down a R-CO-carboxylic component is high as it is the weakest point of the molecular structure of esters-type synthetic oils. These carboxylated products can act as emulsifiers and potentially emulsify themselves to form micelles resulting in retention of liquids in the abdominal cavity of the exposed fish. It is not known if this condition is reversible. A chemical characterization of the liquid collected in the fish’s cavity would prove or disprove the presence of carboxylated products as causative agents. Such analysis was not performed during this initial study however the fact that only ester-exposed fish developed the condition points at the ester synthetic oil as the responsible chemical, as all SBMs tested had similar formulation except for the base fluid.

SBMs can be composed of several ingredients including the base oil, barite and/or bentonite, viscosifiers, emulsifiers, etc. Fish have been exposed to the formulated drilling muds as it would be used during drilling operations. Except for an increase CF, seemingly related to ester-based muds, it is not known which ingredients triggered which biomarker as chemical interactions between individual ingredients can occur within the product, and assimilation of waterborne chemicals by the fish can lead to transformations. In fact, studies have measured effects of individual SBM components, and have substituted the biologically active ingredients for more acceptable substitutes resulting in more environmentally acceptable drilling muds [4], [33].

It is recognized that exposure of fish to drilling muds in a semi-static environment results in an unrealistic exposure level to weathering drilling muds. It has to be kept in mind that this initial experiment is not an attempt to replicate environmental conditions but rather, represents a first effort at investigating potential effects of synthetic-based drilling muds on fish health. To our knowledge, no other study has reported physiological and biomarker changes in fish following exposure to synthetic-based drilling fluids. Results of such chronic exposure can contribute additional information on the assessment of the environmental performance of specific drilling fluids. In the field, fish are attracted to cutting piles as the crushed rock piles create a new environment where fish communities can establish themselves. While exposure concentration in a field situation would likely be lower than the one tested in the laboratory, exposure time would be longer which might result in similar outcomes for the fish.

Under field condition, cutting piles would contain petroleum hydrocarbons adhering to the drilling muds from the oil and gas formation being drilled. Whilst petroleum hydrocarbons generally have a low water solubility, fish biomarkers are sensitive enough to be triggered by very low exposure levels. In a case where fish would be exposed to cutting piles containing petroleum hydrocarbons, the suite of biomarkers would reflect exposure to both drilling muds and to petroleum hydrocarbons. The results reported in this paper provide a baseline for exposure to drilling muds alone, and a different biomarker induction pattern under field conditions could be related to exposure to petroleum hydrocarbons. The suite of biomarkers selected for this research has been identified as the most relevant to exposure to petroleum hydrocarbons [8].

Recent research has demonstrated that biomarkers of exposure and effects to petroleum hydrocarbons have to potential to return to baseline levels following cessation of exposure. Following the 70-days Montara oil spill in the Timor Sea in 2009, fish demonstrated a trend towards baseline levels within one year [34] and have returned to reference levels within two years [35]. In a case where fish would be exposed to petroleum hydrocarbons adhering to the drilling muds, it could be expected that fish biomarkers would indicate chronic exposure as degradation of drilling muds and petroleum hydrocarbons is restricted to the few top millimeters of the cutting piles where aerobic degradation occurs [3], [36].

Ester-based class of synthetic drilling muds has developed a reputation in the petroleum exploration industry as “green chemicals’’ due to their rapid biodegradation rates under anaerobic conditions [1]. Industry often pays a premium for the use of ester-based muds, with the goal to reduce environmental impact through the rapid degradation of the SBM. Biodegradation rate might be a desirable characteristic for specific receiving environments. However, a rapid biodegradation rate might be adverse to certain environments. The results of the present study have shown that exposure to weathering ester-based muds caused the most effects on fish health and consequently, the discharge of ester-based muds might not be desirable in an environment where long-lived fish or other sensitive ecological compartments would be exposed to rapidly degrading ester-based muds. Obviously, the selection of a mud by a drilling operator will consider other factors such as technical performance and drilling conditions. The characteristics of the receiving environment should be of prime importance in the selection of a SBM to be discharged, as some environments might be able to absorb a strong impact over a short time (e.g. a rapidly degrading SBM) while other environments would cope better with a weaker effect over a longer period (e.g. from a slowly degrading SBM). In Western Australia, fish biomarker studies such as the one described here are used by environmental regulators and managers to select specific drilling muds, along with other considerations such as acute toxicity data, SBM technical properties, and the characteristics of the receiving environment.
